# A Next-Generation Sequencing-Based Platform for Quantitative Detection of Hepatitis B Virus Pre-S Mutants in Plasma of Hepatocellular Carcinoma Patients

**DOI:** 10.1038/s41598-018-33051-4

**Published:** 2018-10-04

**Authors:** Chiao-Fang Teng, Hsi-Yuan Huang, Tsai-Chung Li, Woei-Cherng Shyu, Han-Chieh Wu, Chien-Yu Lin, Ih-Jen Su, Long-Bin Jeng

**Affiliations:** 1 Graduate Institute of Biomedical Sciences, China Medical University, Taichung, Taiwan; 2 Organ Transplantation Center, China Medical University Hospital, Taichung, Taiwan; 3 Department of Laboratory Medicine, China Medical University Hospital, Taichung, Taiwan; 4 Department of Public Health, College of Public Health, China Medical University, Taichung, Taiwan; 5 Department of Healthcare Administration, College of Medical and Health Science, Asia University, Taichung, Taiwan; 6 Translational Medicine Research Center and Department of Neurology, China Medical University Hospital, Taichung, Taiwan; 7 Department of Occupational Therapy, Asia University, Taichung, Taiwan; 8 National Institute of Infectious Diseases and Vaccinology, National Health Research Institutes, Tainan, Taiwan; 9 Graduate Institute of Clinical Medical Science and School of Medicine, China Medical University, Taichung, Taiwan; 10 Department of Laboratory Medicine, Taichung Tzu Chi Hospital, Buddhist Tzu Chi Medical Foundation, Taichung, Taiwan; 11 Department of Biotechnology, Southern Taiwan University of Science and Technology, Tainan, Taiwan; 12 Department of Pathology, National Cheng Kung University Hospital, Tainan, Taiwan

## Abstract

Hepatocellular carcinoma (HCC) is a leading cause of cancer-related death worldwide. Early diagnosis and treatment of HCC remain a key goal for improving patient survival. Chronic hepatitis B virus (HBV) infection is a major risk factor for HCC development. Pre-S mutants harboring deletions in HBV large surface antigen have been well demonstrated as HBV oncoproteins that dysregulate multiple signaling pathways in hepatocytes, leading to HCC formation. The presence of pre-S mutants in plasma represents important predictive and prognostic markers for HCC in patients with chronic HBV infection. However, the method to detect pre-S mutants remains to be optimized. In this study, we developed a platform, based on the next-generation sequencing (NGS) technology, for detection of pre-S mutants in plasma of HBV-related HCC patients. Compared to the current TA cloning-based analysis, the NGS-based analysis could detect pre-S deletion quantitatively, and the detection rate was significantly more sensitive in 49 plasma analyzed (McNemar’s paired proportion test, P value < 0.0001; simple kappa coefficient, κ = 0.29 (95% CI, 0.12 to 0.46)). Our data suggest that the NGS-based platform may hold a promise for improving the clinical application of pre-S mutants in serving as predictive and prognostic markers for HBV-related HCC.

## Introduction

Hepatocellular carcinoma (HCC) is the sixth most common cancer and the third leading cause of cancer-related death worldwide, with an estimated 500,000 deaths per year^1–3^. Although surgical resection is regarded as a potentially curable treatment for HCC patients, majority of HCC patients are not medically suitable for this treatment and HCC recurrence after surgery remains a frequent event, leading to poor patient survival^4,5^. Liver transplantation is well established as a therapeutic treatment for patients with unresectable HCC but the scarcity of donors limits this treatment^6^. Many nonsurgical therapeutic options are also available for HCC patients, whereas these treatments exhibit limited survival benefit^7,8^. Therefore, early diagnosis and treatment of HCC remain a key goal for improving patient survival.

**Figure 1:**
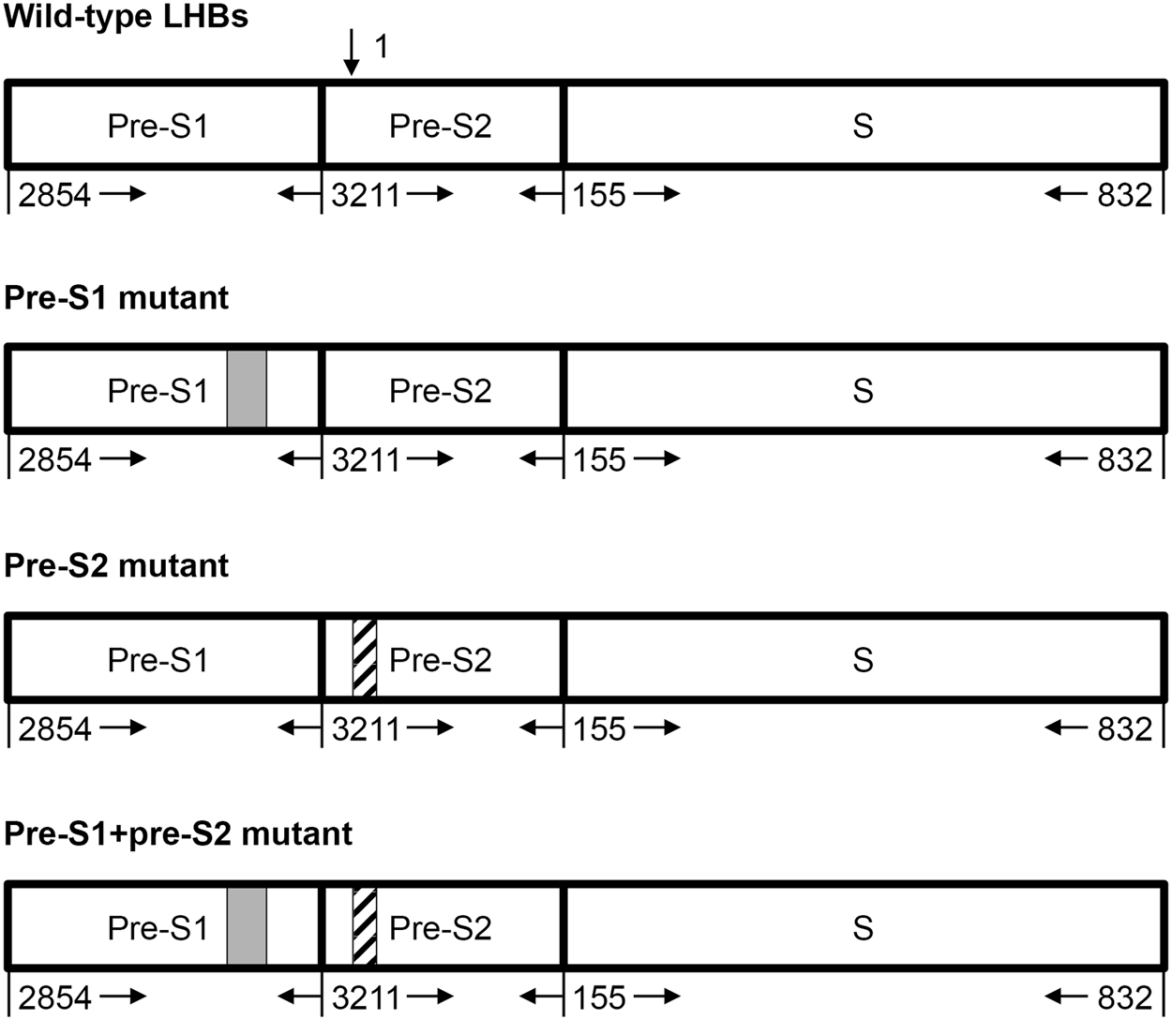
Schematic representation of the wild-type and pre-S mutant LHBs gene. The wild-type LHBs gene contains three intact gene segments, pre-S1, pre-S2, and S. The numbers on the bottom of the gene indicate the nucleotide positions of each gene segment in the HBV genome. The arrow at the top of the diagram indicates the start (nucleotide 1) of the circular genome, and the numbers go clockwise and end at nucleotide 3221 (not shown). Here only the LHBs gene of the genome is shown. The pre-S1, pre-S2, and pre-S1 + pre-S2 mutants contain deletion mutations in the pre-S1, pre-S2, and both pre-S1 and pre-S2 gene segments, respectively. The grey and hatched boxes represent the regions deleted in the pre-S1 and pre-S2 gene segments, respectively.

HCC development is intimately associated with chronic hepatitis B virus (HBV) infection, which accounts for over 50% of total cases worldwide^9,10^. Our previous studies have well demonstrated that pre-S mutants, which contain in-frame deletions in the pre-S1 and/or pre-S2 gene segments of HBV large surface antigen (LHBs) (Fig. 1), are HBV oncoproteins and can initiate multiple endoplasmic reticulum (ER) stress-related signaling pathways, contributing to growth advantages of hepatocytes and eventually HCC formation^11–13^. The presence of pre-S mutants in liver tissues or blood carries a 5-fold higher risk of HCC development in patients with chronic HBV infection^14,15^. The prevalence of pre-S mutants is as high as 60% in HCC patients and significantly associated with HCC recurrence after surgical resection^16–18^. As a result, pre-S mutants have emerged as important predictive markers for HCC risk in chronic HBV carriers as well as powerful prognostic markers for recurrence risk in HBV-related HCC patients following surgical resection^14,15,18–20^.

Several methods have been applied to detect the presence of pre-S mutants in chronic HBV carriers and HBV-related HCC patients, including the methods based on immunohistochemistry (IHC) staining of HBV surface antigen (HBsAg)^13,19^, TA cloning and DNA sequencing following polymerase chain reaction (PCR) amplification of pre-S gene (including both pre-S1 and pre-S2 regions)^14–16^, and the usage of Pre-S Gene Chip^17,20^. Hepatocytes harboring pre-S1 and pre-S2 mutants have been shown to manifest themselves histologically as two different types of ground glass hepatocytes (GGHs, designated types I and II, respectively) in liver tissues^13,19^. Following IHC staining, types I and II GGHs express distinct inclusion-like and marginal patterns of HBsAg, respectively, and thus can be distinguished from hepatocytes harboring wild-type LHBs, which express a diffuse and homogeneous pattern of HBsAg^13,19^. In addition, pre-S gene can be specifically amplified from virus DNA extracted from patient’s liver tissue or blood by using nested PCR^14–16^. To detect pre-S deletion, the resulting pre-S gene PCR products either are directly subjected to DNA sequencing (when only a single PCR band is seen in agarose gel) or are first cloned into a TA vector followed by DNA sequencing (when two or more PCR bands are revealed)^14–16^. Alternatively, the pre-S gene PCR products can be analyzed by Pre-S Gene Chip^17,20^. In the cases of only a single PCR band, the pre-S gene PCR products are directly subjected to chip hybridization; in the cases of multiple PCR bands, TA cloning is first performed, followed by PCR amplification of pre-S insert-DNA, chip hybridization and eventually signal development^17,20^.

Although the current methods are successfully used to detect pre-S mutants, they can still only provide qualitative and semi-quantitative detection results. Moreover, since TA cloning is majorly dependent on PCR bands that are clearly separated and visible in agarose gel, the TA cloning-based detection of pre-S mutants may have the risk to omit unseparated or invisible PCR bands, resulting in lower detection sensitivity. Therefore, development of a method that can quantitatively detect pre-S mutants with higher sensitivity and fidelity may improve the clinical application of pre-S mutants in serving as predictive and prognostic markers for HBV-related HCC. In this study, we established a next-generation sequencing (NGS)-based platform for quantitative detection of pre-S mutants in plasma of HBV-related HCC patients. Our data showed that the NGS-based analysis was more sensitive than the TA cloning-based analysis in detection of HBV pre-S mutants.

## Results

### Patient profile and clinicopathological data

The clinicopathological characteristics of the 49 HBV-related HCC patients enrolled in this study are summarized in Table 1. There were 43 (88%) men and 6 (12%) women, and the median age of all patients was 57 years (range, 33 to 78). Forty-three (88%) patients were HBV e antigen (HBeAg) negative. HBV DNA was detected in 27 (55%) patients at a median of 1.4 × 10^5^ copies/mL (range, 30.1 to 1.5 × 10^8^). Tumor size was recorded for 48 (98%) patients (median: 3.5 cm; range: 1.5 to 35 cm).

**Table 1:** Clinicopathological characteristics of the 49 HBV-related HCC patients enrolled in this study (continued).

Characteristics^a^	No. of Patients	Median (Range)
Age (years)	49	57 (33–78)
Gender (men/women)	43/6	
Smoking (yes/no)	20/29	
Alcohol (yes/no)	10/39	
HBeAg (positive/negative)	6/43	
HBV DNA (copies/mL) (20−1.7 × 10^8^/<20)^b^	27/6	1.4 × 10^5^ (30.1–1.5 × 10^8^)
Albumin (g/dL)	48	3.9 (2.0–4.9)
AST (U/L)	49	53 (15–290)
ALT (U/L)	48	58 (20–700)
AFP (ng/mL) (≤54000/>54000)^c^	43/5	26.7 (1.4–3266.0)
Tumor size (cm)	48	3.5 (1.5–35.0)
Tumor encapsulation (yes/no)	32/17	
Lymph node involvement (yes/no)	5/44	
Portal vein thrombosis (yes/no)	2/47	
Satellite nodule (yes/no)	8/41	
Vascular invasion (microscopic/macroscopic/no)	19/2/28	
Distant metastasis (yes/no)	3/46	
Steatosis grade (0/1/2/3)	39/9/1/0	
Metavir inflammation score (0/1/2/3)	4/38/3/0	
Ishak fibrosis score (0/1/2/3/4/5/6)	0/4/11/10/21/1/1	
Child-Pugh cirrhosis score (A/B/C)	32/12/4	
CLIP score (0/1/2/3/4/5/6)	16/18/9/2/2/0/1	
BCLC stage (A/B/C/D)	32/9/5/3	
AJCC TNM stage (I/II/IIIA/IIIB/IIIC/IVA/IVB)	24/14/4/1/5/0/1	

^a^Only patients with available data were analyzed.

^b^HBV DNA was measured with a range of 20 to 1.7 × 10^8^ copies/mL.

^c^AFP was measured with the highest detection limit of 54000 ng/mL.

Abbreviations: HBV, hepatitis B virus; HCC, hepatocellular carcinoma; HBeAg, hepatitis B e antigen; AST, aspartate aminotransferase; ALT, alanine aminotransferase; AFP, alpha-fetoprotein; CLIP, Cancer of the Liver Italian Program; BCLC, Barcelona Clinic Liver Cancer; AJCC, American Joint Committee on Cancer; TNM, tumor-node-metastasis.

### Pre-S genotype and patient grouping

According to the pre-S gene segments in which deletion mutations took place, the pre-S deletion fell into three types, the pre-S1, pre-S2, and pre-S1 + pre-S2 deletion, which spanned the pre-S1, pre-S2, and both pre-S1 and pre-S2 gene segments, respectively (Fig. 1). Therefore, each patient could be classified into one of the following pre-S genotype groups: the patients without pre-S deletion (wild-type), and the patients with pre-S deletion, including the patients with any one or two or all three types of pre-S deletion. Alternatively, the patients with pre-S deletion could be classified into two groups, simply depending on either pre-S1 or pre-S2 gene segment containing deletion mutations.

### TA cloning-based pre-S genotyping in plasma of HBV-related HCC patients

By using TA cloning-based pre-S genotyping analysis, we first detected pre-S deletion in plasma of the 49 HBV-related HCC patients. As shown in Supplementary Table S1 and Table 2, pre-S deletion was detected in 17 out of 49 (35%) patients, among whom 9 (19%) patients had only pre-S1 deletion, 4 (8%) patients had only pre-S2 deletion, 3 (6%) patients had only pre-S1 + pre-S2 deletion, and 1 (2%) patient had both pre-S1 and pre-S1 + pre-S2 deletion. Additionally, 13 (27%) and 8 (16%) patients had deletion spanning the pre-S1 and pre-S2 gene segments, respectively. Each pre-S deletion region was also defined.

**Table 2:** Summary of the pre-S genotyping results by TA cloning- and NGS-based analyses in 49 HBV-related HCC patients.

Summary of the Pre-S Genotyping Results	TA Cloning-Based Analysis	NGS-Based Analysis
		cut-off percentage: 5.049
Total patients (n) (%)	49 (100)	49 (100)
Patients without pre-S del (n) (%)	32 (65)	12 (25)
Patients with pre-S del (n) (%)	17 (35)	37 (75)
Patients with only pre-S1 del (n) (%)	9 (19)	10 (20)
Patients with only pre-S2 del (n) (%)	4 (8)	5 (10)
Patients with only pre-S1 + pre-S2 del (n) (%)	3 (6)	0 (0)
Patients with both pre-S1 and pre-S2 del (n) (%)	0 (0)	6 (12)
Patients with both pre-S1 and pre-S1 + pre-S2 del (n) (%)	1 (2)	0 (0)
Patients with both pre-S2 and pre-S1 + pre-S2 del (n) (%)	0 (0)	1 (2)
Patients with all three types of pre-S del (n) (%)	0 (0)	15 (31)
Patients with deletion spanning pre-S1 gene segment (n) (%)	13 (27)	32 (65)
Patients with deletion spanning pre-S2 gene segment (n) (%)	8 (16)	27 (55)

Abbreviations: del, deletion; n, number.

### NGS-based analysis detected pre-S deletion quantitatively

Next, we developed a NGS-based platform to detect pre-S deletion in plasma of the 49 HBV-related HCC patients. As shown in Supplementary Table S1, the same as the TA cloning result, the NGS result provided information on not only the pre-S deletion type but also the detailed pre-S deletion region. Importantly, in contrast to the TA cloning-based analysis, NGS-based analysis detected pre-S deletion quantitatively so that the frequency of each pre-S gene DNA within the pre-S gene PCR products could be determined. As a result, all three types of pre-S deletion could be detected in plasma of each patient although the frequency was as low as one copy of DNA in the analyzed PCR product. Notably, when the pre-S gene DNA with the highest frequency in each type of pre-S deletion was selected, its deletion region perfectly coincided with that of the same type of pre-S deletion clone detected by TA cloning in all analyzed patients.

By matching the result of TA cloning with that of NGS, the corresponding pre-S gene DNA detected by both analyses had the lowest frequency of 5.049%, as shown by the wild-type pre-S gene DNA in the patient No. 37 (Supplementary Table S1). Therefore, we set the frequency of 5.049% as the cut-off percentage value to divide the patients analyzed by NGS into one of the pre-S genotype groups. As shown in Table 2, up to 37 of 49 (75%) patients had pre-S deletion, among whom 10 (20%) patients had only pre-S1 deletion, 5 (10%) patients had only pre-S2 deletion, 6 (12%) patients had both pre-S1 and pre-S2 deletion, 1 (2%) patient had both pre-S2 and pre-S1 + pre-S2 deletion, and 15 (31%) patients had all three types of pre-S deletion. Moreover, up to 32 (65%) and 27 (55%) patients had deletion spanning the pre-S1 and pre-S2 gene segments, respectively.

### NGS was more sensitive than TA cloning for detection of pre-S deletion in plasma of HBV-related HCC patients

By comparing the results between TA cloning and NGS in total 49 patients, pre-S deletion was detected in 17 (35%) and 37 (75%) patients by TA cloning- and NGS-based analyses, respectively (Table 2). Up to 20 (40%) patients were negative for pre-S deletion by TA cloning-based analysis but conversely were positive for pre-S deletion by NGS-based analysis. The detection of pre-S deletion was significantly more sensitive by NGS- than by TA cloning-based analysis (McNemar’s paired proportion test, P value  < 0.0001) (Table 3). Also, the simple kappa value for the agreement between both analyses in detection of pre-S deletion was fair at 0.29 (95% confidence interval (CI), 0.12 to 0.46) (Table 3). Similar result could be observed when comparing the concordance between both analyses in detection of deletion spanning the pre-S1 (McNemar’s paired proportion test, P value  < 0.0001; simple kappa coefficient, kappa value (κ) = 0.32 (95% CI, 0.15 to 0.49)) or pre-S2 (McNemar’s paired proportion test, P value  < 0.0001; simple kappa coefficient, κ = 0.27 (95% CI, 0.09 to 0.45)) gene segment (Table 3).

**Table 3:** Comparison of the pre-S genotyping results by TA cloning- and NGS-based analyses in 49 HBV-related HCC patients.

		McNemar’s paired proportion test			Simple Kappa Coefficient	
		NGS (−)	NGS (+)	P value	κ value	95% CI
Patients without (−)/with (+) pre-S del (n) (%)	TA cloning (−)	12 (24)^a^	20 (40)^b^	<0.0001	0.29	0.12–0.46
	TA cloning (+)	0 (0)^c^	17 (34)^d^			
Patients without (−)/with (+) deletion	TA cloning (−)	17 (34)	19 (38)	<0.0001	0.32	0.15–0.49
spanning pre-S1 gene segment (n) (%)	TA cloning (+)	0 (0)	13 (26)			
Patients without (−)/with (+) deletion	TA cloning (−)	22 (44)	19 (38)	<0.0001	0.27	0.09–0.45
spanning pre-S2 gene segment (n) (%)	TA cloning (+)	0 (0)	8 (16)			

^a^The number of patients without the indicated pre-S deletion by both TA cloning- and NGS-based analyses.

^b^The number of patients with the indicated pre-S deletion by NGS- but not TA cloning-based analysis.

^c^The number of patients with the indicated pre-S deletion by TA cloning- but not NGS-based analysis.

^d^The number of patients with the indicated pre-S deletion by both TA cloning- and NGS-based analyses.

Abbreviations: n, number.

**Figure 2:**
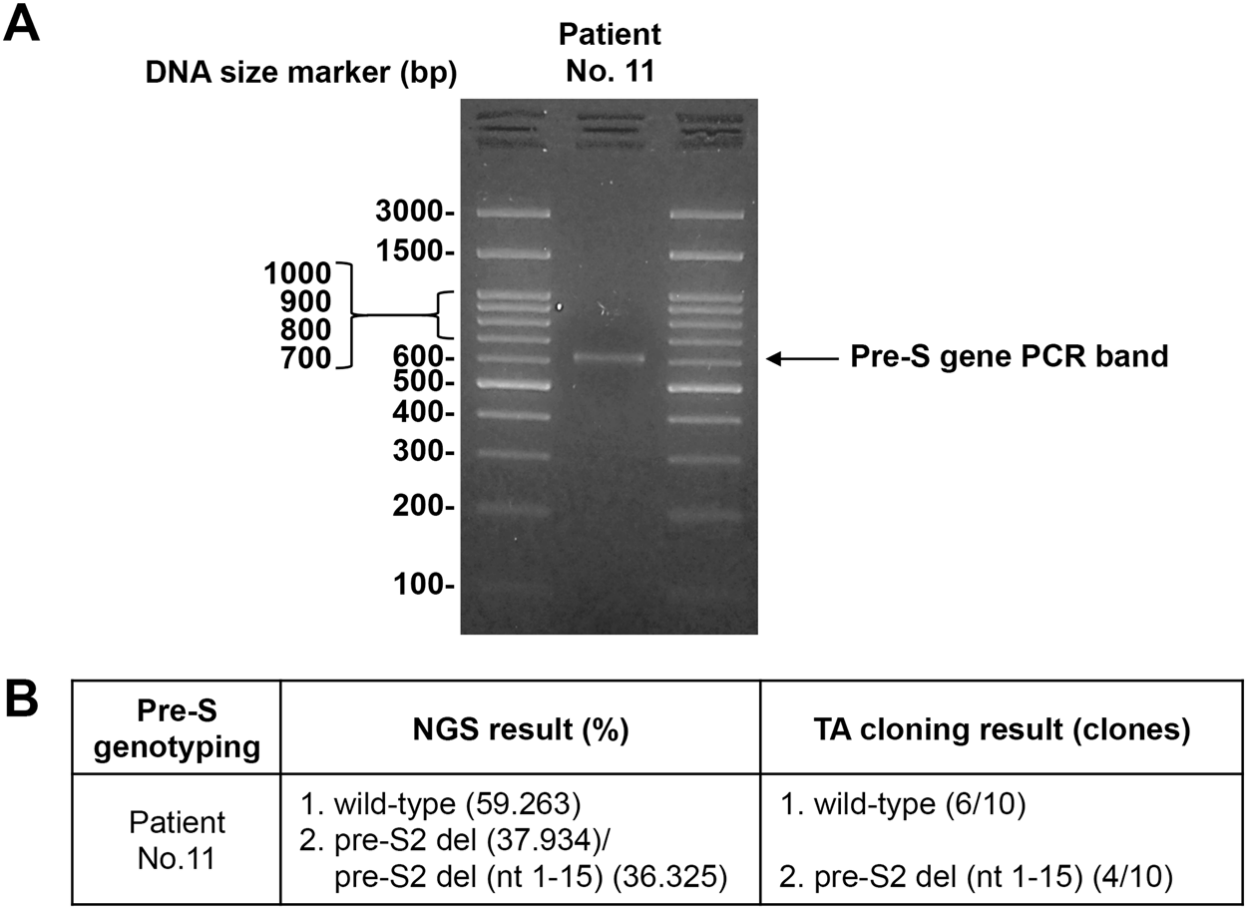
The pre-S genotyping result by additional TA cloning-based analysis in selected HBV-related HCC patient. (**A**) The pre-S gene PCR products prepared from the plasma DNA of the selected patient No. 11 were resolved by agarose gel electrophoresis with the 100-bp DNA size marker loaded on both sides. The single pre-S gene PCR band was indicated by arrow. The size of the marker was shown in base pairs. (**B**) Following TA cloning of the single pre-S gene PCR band, 10 plasmid clones containing the pre-S insert-DNA were randomly selected for Sanger DNA sequencing analysis. The result was shown as the ratio of the number of clones containing either wild-type or deletion pre-S gene DNA to the total number of clones sequenced. The NGS result was shown for comparison. For simplicity, only the pre-S wild-type or deletion whose total frequency was above the cut-off percentage value was shown. The pre-S gene DNA with the highest frequency in the indicated type of pre-S deletion and its deletion region was also shown. Abbreviations: bp, base pairs; del, deletion; nt, nucleotide.

Because the PCR bands that are too close in size are difficult to be clearly separated by agarose gel electrophoresis, these bands might be visualized together as a single band in agarose gel. In such cases, when the TA cloning-based pre-S genotyping was performed, the individual pre-S gene PCR bands that remained unseparated as a single band in agarose gel might be possibly randomly omitted from analysis during the cloning process, resulting in the lower detection sensitivity of pre-S deletion. To clarify this possibility, we selected the patient No. 11 from the 50 HBV-related HCC patients for further analysis. Based on the result of TA cloning, this patient was negative for pre-S deletion (Supplementary Table S1). However, the result of NGS showed that this patient had pre-S2 deletion, and especially, the deletion pre-S gene DNA with the highest frequency was very close in size (only 15 base pairs in difference) with the wild-type pre-S gene DNA (Supplementary Table S1). As shown in Fig. 2, we carried out an additional TA cloning-based pre-S genotyping analysis for this patient, in which the single pre-S gene PCR band visualized in agarose gel was cloned into the TA vector, and 10 plasmid clones containing the pre-S insert-DNA were then randomly selected for Sanger DNA sequencing analysis. We observed that the 10 randomly selected plasmid clones of this patient contained either wild-type or deletion pre-S gene DNA, and the pre-S deletion region coincided with that by NGS analysis. This data might partly explain the lower detection sensitivity of pre-S deletion by TA cloning- than by NGS-based analysis.

## Discussion

Despite remarkable progress in developing therapies for HCC, the high morbidity and mortality rates of HCC in patients with chronic HBV infection remain a big challenge^21,22^. Overall, liver resection and transplantation are still the best therapeutic options for patients with HCC^5^. However, the former is suitable for the patients with single or smaller HCC, and the latter is limited by the shortage of donor livers^4,6^. Early diagnosis of HCC is thus urgently needed for early treatment of HCC by surgical resection or allowing enough time for the patients waiting for suitable donor livers, improving the patient survival. Pre-S mutants, which harbor deletion mutation in the pre-S gene segments of LHBs, have been demonstrated as HBV oncoproteins that disturb a variety of ER stress-related signaling pathways in hepatocytes, contributing to genomic instability and HCC development^11–13^. It has been well documented that the presence of pre-S mutants in plasma represents a predictive marker for HCC risk in chronic HBV carriers as well as a prognostic marker for recurrence risk in HBV-related HCC patients following surgical resection^14,15,18–20^. Therefore, development of a method that can detect pre-S mutants with high sensitivity and accuracy may improve the clinical application of pre-S mutants in serving as predictive and prognostic markers for HBV-related HCC, providing survival benefit for the patients.

To date, besides the IHC-based method^13,19^, the methods applied to detect pre-S mutants in blood or liver tissues of patients with chronic HBV infection or HBV-related HCC are mainly based on the TA cloning approach^14–17,20^. Following PCR amplification of pre-S gene from the patient specimen, TA cloning approach is well established for isolation of specific pre-S gene DNA from the PCR products for further analysis. However, this approach has the following drawbacks in practical operation. First, the cloning procedure is somewhat time-consuming, especially in cases where multiple PCR bands of variable intensity are visualized in agarose gel. Moreover, the methodology is largely dependent on the visualization of PCR bands in agarose gel. The PCR bands with intensity below the detection limit cannot be visualized in agarose gel for the cloning. Last but not least, there is a difficult in clearly separating the PCR bands that are too close in size by agarose gel electrophoresis. As shown in this study, we demonstrated that when the wild-type and deletion pre-S gene DNA were too close in size to be clearly separated in agarose gel, the deletion pre-S gene DNA could be possibly omitted from detection during the cloning process, thus decreasing the detection sensitivity and increasing the false-negative rate by TA cloning-based pre-S genotyping analysis.

Over the past decade, the invention of NGS technology has revolutionized almost all fields of genomic studies^23–27^. In contrast to traditional Sanger DNA sequencing^28,29^, NGS technology performs massively parallel sequencing, during which millions of fragments of DNA from a single sample are sequenced in unison, providing qualitative and quantitative information on the sequence and number of individual DNA fragments at an incredible throughput^30,31^. As a result, for NGS there is no need to carry out TA cloning for individual DNA molecules before the sequencing analysis. Therefore, in this study, based on the NGS technology we developed a new method for detection of pre-S mutants in plasma of HBV-related HCC patients. Compared to the current TA cloning-based methods, our data showed that the NGS-based method exhibited several advantages. First, the sample preparation before analysis was more simple and easy. The pre-S gene PCR products could be directly subjected to NGS analysis without the steps of agarose gel electrophoresis and TA cloning. Additionally, individual pre-S gene DNA molecules were detected qualitatively and quantitatively so that their deletion region and frequency within the PCR products could be well determined. Most important of all, the detection of pre-S deletion was more sensitive. There were no concerns on the detection and resolution limitations by agarose gel electrophoresis. As low as one copy of pre-S gene DNA in the PCR products could be detected without the risk of omitting due to either the DNA intensity being too low to be visualized or the DNA size being too close to others to be clearly separated.

In conclusion, in this study we developed a NGS-based platform for detection of HBV pre-S mutants in plasma of HCC patients. Compared to the current TA cloning-based methods, our data showed that the NGS-based method could detect pre-S deletion quantitatively with a higher sensitivity, suggesting that the NGS-based platform may provide better accuracy for detection of pre-S deletion, potentially improving the outcome prediction of patients with HBV-related HCC.

## Methods

### Human plasma samples

In this study, the plasma samples were obtained retrospectively from 49 HBV-related HCC patients at China Medical University Hospital (Taichung, Taiwan), from July 2008 to May 2017, under the approval of the China Medical University & Hospital Research Ethics Committee. All plasma samples were stored at −80 °C for TA cloning- and NGS-based pre-S genotyping. The clinicopathological records of the patients were also collected. All research was performed in accordance with relevant guidelines and regulations and the informed consent was obtained from all participants.

### TA cloning-based pre-S genotyping analysis

**Figure 3:**
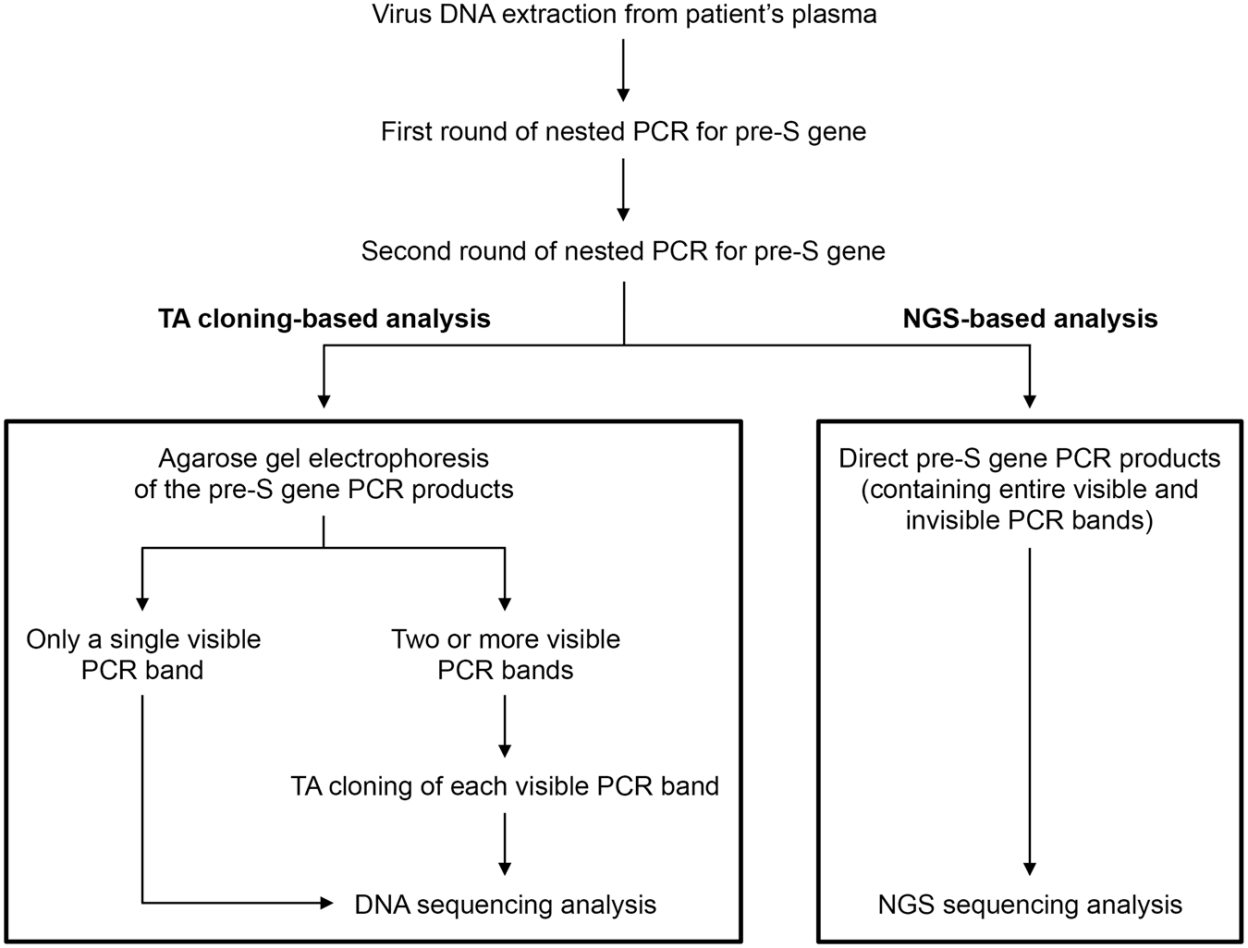
Flowchart of the pre-S genotyping by TA cloning- and NGS-based analysis. The virus DNA is extracted from patient’s plasma. The pre-S gene is amplified from virus DNA following first and second rounds of nested PCR. For TA cloning-based analysis, the pre-S gene PCR products are visualized by using agarose gel electrophoresis. When only a single PCR band is seen in agarose gel, the products are directly subjected to DNA sequencing analysis. However, when two or more PCR bands are revealed, individual bands are first cloned into the TA vector for further DNA sequencing analysis. In contrast, for NGS-based analysis, the entire pre-S gene PCR products, including both visible and invisible bands, are directly subjected to NGS sequencing analysis without the complex procedures needed for TA cloning-based analysis.

For TA-cloning-based detection of pre-S deletion, 200 μL of plasma of each patient was used for DNA extraction with the DNeasy Blood Kit (Qiagen, Valencia, CA) according to the manufacturer’s instructions. The pre-S gene was amplified from the plasma DNA, which contains virus DNA, by using nested PCR with primers for two successive rounds of reactions following the program shown in Table 4. The nested PCR was performed with the high-fidelity Platinum SuperFi DNA polymerase (Invitrogen, Carlsbad, CA), and the resulting pre-S gene PCR products were resolved by agarose gel electrophoresis. As shown in Fig. 3, in cases where only a single PCR band was visualized, the pre-S gene PCR products were directly subjected to Sanger DNA sequencing analysis. In cases where multiple PCR bands (approximately ranging from 350 to 600 base pairs in size) were visualized, individual bands in agarose gel were separately excised, purified, and cloned using the TOPO TA Cloning Kit (Invitrogen) after conducting A-tailing procedure, and the resulting plasmid clones containing the pre-S insert-DNA were then directed to Sanger DNA sequencing analysis.

**Table 4:** The primers and program of nested PCR for amplification of HBV pre-S gene.

Primer	Sequence (5′ to 3′)	Position on the HBV genome
Pre-S-1F Pre-S-1R Pre-S-2R	GCGGGTCACCATATTCTTGGG GAGTCTAGACTCTGCGGTAT TAACACGAGCAGGGGTCCTA	nt 2818 to 2837 nt 236 to 255 nt 180 to 199
**Nested PCR**	**Primer pairs**	**Program**
First round	Pre-S-1F & Pre-S-1R	Stage 1 95 °C, 1 min Stage 2 (35 cycles) 95 °C, 1 min 55 °C, 1 min 72 °C, 1 min Stage 3 72 °C, 7 min
Second round	Pre-S-1F & Pre-S-2R	Stage 1 98 °C, 1 min Stage 2 (35 cycles) 98 °C, 1 min 58 °C, 1 min 72 °C, 1 min Stage 3 72 °C, 7 min

Abbreviations: nt, nucleotide.

### NGS-based pre-S genotyping analysis

For NGS-based analysis of pre-S deletion, the pre-S gene was amplified from the plasma DNA of each patient by using the same condition of nested PCR as the TA cloning-based analysis (Table 4). Unlike TA cloning-based analysis, the pre-S gene PCR products, regardless of containing single or multiple visible, even invisible bands in agarose gel, were directly subjected to NGS sequencing analysis without the need for gel electrophoresis, band excision, purification, and cloning (Fig. 3). For NGS sequencing analysis, DNA libraries were prepared using standard KAPA LTP Library Preparation Kit for Illumina Platforms (Kapa Biosystems, Boston, MA). Base calling and quality scoring were performed by an updated implementation of Real-Time Analysis on the NextSeq 500 system (Illumina, San Diego, CA) according to the manufacturer’s instructions. The bcl2fastq Conversion Software v2.20 (Illumina) was used to demultiplex data and convert BCL files to standard FASTQ file formats. After trimming the adaptor sequence and masking the low-complexity sequence with a quality score under 20, the sequence reads were compared with the master sequences from the reference sets of HBV genotypes, which were available on the NCBI website (https://www.ncbi.nlm.nih.gov/projects/genotyping/view.cgi?db=2), by using BLAST. The E-value cutoff was set as 1e-5 to reduce false-positive result. Finally, the pre-S deletion type, region, and frequency were determined by using our customized scripts. The deletion frequency was defined as the counts of reads with deletion divided by the total number of reads and then multiplied by 100.

### Statistical analysis

The McNemar’s paired proportion test was used to compare the difference between TA cloning- and NGS-based analyses for detection of pre-S deletion, where a P value < 0.05 was considered to indicate statistical significance. Also, the agreement between both analyses in pre-S genotyping was evaluated by calculating the simple kappa coefficient with 95% CI, where κ = 1 denotes perfect agreement; κ > 0.80, excellent; κ > 0.60, good; κ > 0.40, moderate; κ > 0.20, fair; and κ > 0 indicates poor agreement^32^.

## Electronic supplementary material

Supplementary Table S1

## Data Availability

All data generated or analyzed during this study are included in this published article and its supplementary information files.
