# Supplementary material for: A Next-Generation Sequencing-Based Platform for Quantitative Detection of Hepatitis B Virus Pre-S Mutants in Plasma of Hepatocellular Carcinoma Patients
Source: Sci Rep. 2018 Oct 4;8:14816. doi: 10.1038/s41598-018-33051-4 (PMC6172208; doi:10.1038/s41598-018-33051-4)
Supplement: Supplementary file 1 — Supplementary Table S1 [file 41598_2018_33051_MOESM1_ESM.pdf]

A Next-Generation Sequencing-Based Platform for Quantitative Detection of Hepatitis B Virus Pre-S Mutants in Plasma of Hepatocellular Carcinoma Patients

Chiao-Fang Teng,[1,2*] Hsi-Yuan Huang,[3] Tsai-Chung Li,[4,5] Woei-Cherng Shyu,[1,6,7] Han-Chieh Wu,[8] Chien-Yu Lin,[3,9] Ih-Jen Su,[7,10,11*] and Long-Bin Jeng[2*]

[1]Graduate Institute of Biomedical Sciences, China Medical University, Taichung, Taiwan
[2]Organ Transplantation Center, China Medical University Hospital, Taichung, Taiwan
[3]Department of Laboratory Medicine, China Medical University Hospital, Taichung, Taiwan
[4]Department of Public Health, College of Public Health, China Medical University, Taichung, Taiwan
[5]Department of Healthcare Administration, College of Medical and Health Science, Asia University, Taichung, Taiwan
[6]Translational Medicine Research Center and Department of Neurology, China Medical University Hospital, Taichung, Taiwan
[7]Department of Occupational Therapy, Asia University, Taichung, Taiwan
[8]National Institute of Infectious Diseases and Vaccinology, National Health Research Institutes, Tainan, Taiwan
[9]Graduate Institute of Clinical Medical Sciences, China Medical University, Taichung, Taiwan
[10]Department of Biotechnology, Southern Taiwan University of Science and Technology, Tainan, Taiwan
[11]Department of Pathology, National Cheng Kung University Hospital, Tainan, Taiwan

[*]Long-Bin Jeng, Ih-Jen Su, and Chiao-Fang Teng are corresponding authors

Supplementary Table 1. List of the pre-S genotyping results by TA cloning- and NGS-based analyses in 49 HBV-related HCC patients

Patient No.	TA Cloning Result[a]	NGS Result (Pre-S Deletion Type (%))[b]	NGS Result (Pre-S Deletion Region (%))[c]
1	pre-S1 del (nt 2855-2872)	1. **pre-S1 del (92.118)**[d]<br>**2. wild-type (7.278)**<br>3. pre-S2 del (0.372)<br>4. pre-S1+pre-S2 del (0.231)	1. pre-S1 del (nt 2855-2872) (90.102)<br>2. wild-type (7.278)<br>3. pre-S2 del (nt 1-28) (0.180)<br>4. pre-S1+pre-S2 del (nt 2855-2872, 1-111) (0.027)
2	1. wild-type<br>2. pre-S1 del (nt 2910-3089)	1. **pre-S1 del (75.241)**<br>2. **wild-type (22.338)**<br>3. pre-S2 del (1.891)<br>4. pre-S1+pre-S2 del (0.530)	1. pre-S1 del (nt 2910-3089) (48.977)<br>2. wild-type (22.338)<br>3. pre-S2 del (nt 1-54) (1.579)<br>4. pre-S1+pre-S2 del (nt 2910-3089, 1-57) (0.277)
3	wild-type	1. **pre-S1 del (76.228)**<br>2. **wild-type (12.583)**<br>3. **pre-S2 del (10.622)**<br>4. pre-S1+pre-S2 del (0.567)	1. pre-S1 del (nt 3110-3127) (24.869)<br>2. wild-type (12.583)<br>3. pre-S2 del (nt 1-57) (10.000)<br>4. pre-S1+pre-S2 del (nt 2855-2872, 1-57) (0.490)
4	wild-type	1. **wild-type (71.590)**<br>2. **pre-S1 del (28.270)**<br>3. pre-S2 del (0.129)<br>4. pre-S1+pre-S2 del (0.011)	1. wild-type (71.590)<br>2. pre-S1 del (nt 2854-3147) (23.961)<br>3. pre-S2 del (nt 3211-3216) (0.048)<br>4. pre-S1+pre-S2 del (nt 2954-3097, 1-54) (0.002)
5	pre-S2 del (nt 24-50)	1. **wild-type (58.461)**<br>2. **pre-S2 del (37.194)**<br>3. pre-S1 del (2.939)<br>4. pre-S1+pre-S2 del (1.407)	1. wild-type (58.461)<br>2. pre-S2 del (nt 24-50) (33.801)<br>3. pre-S1 del (nt 2880-3146) (0.646)<br>4. pre-S1+pre-S2 del (nt 2880-3146, 24-50) (0.355)
6	wild-type	1. **wild-type (99.278)**<br>2. pre-S1 del (0.660)<br>3. pre-S2 del (0.053)<br>4. pre-S1+pre-S2 del (0.009)	1. wild-type (99.278)<br>2. pre-S1 del (nt 3100-3129) (0.084)<br>3. pre-S2 del (nt 1-54) (0.009)<br>4. pre-S1+pre-S2 del (nt 2954-3097, 1-15) (0.004)

Supplementary Table 1. List of the pre-S genotyping results by TA cloning- and NGS-based analyses in 49 HBV-related HCC patients (continued)

Patient No.	TA Cloning Result[a]	NGS Result (Pre-S Deletion Type (%))[b]	NGS Result (Pre-S Deletion Region (%))[c]
7	1. wild-type	1. **pre-S1 del (75.109)**	1. pre-S1 del (nt 2854-2970) (65.653)
	2. pre-S1 del (nt 2854-2970)	2. **wild-type (23.879)**	2. wild-type (23.879)
		3. pre-S1+pre-S2 del (0.687)	3. pre-S1+pre-S2 del (nt 2855-2970, 1-2) (0.219)
		4. pre-S2 del (0.325)	4. pre-S2 del (nt 1-54) (0.149)
8	wild-type	1. **pre-S1 del (86.404)**	1. pre-S1 del (nt 3110-3127) (26.821)
		2. **wild-type (12.695)**	2. wild-type (12.695)
		3. pre-S2 del (0.737)	3. pre-S2 del (nt 1-12) (0.219)
		4. pre-S1+pre-S2 del (0.163)	4. pre-S1+pre-S2 del (nt 2855-2872, 1-54) (0.016)
9	wild-type	1. **wild-type (96.592)**	1. wild-type (96.592)
		2. pre-S1 del (2.906)	2. pre-S1 del (nt 3026-3205) (0.604)
		3. pre-S2 del (0.469)	3. pre-S2 del (nt 1-12) (0.233)
		4. pre-S1+pre-S2 del (0.033)	4. pre-S1+pre-S2 del (nt 2854-2979, 6-134) (0.008)
10	wild-type	1. **wild-type (99.000)**	1. wild-type (99.000)
		2. pre-S1 del (0.927)	2. pre-S1 del (nt 3106-3129) (0.294)
		3. pre-S2 del (0.062)	3. pre-S2 del (nt 1-54) (0.031)
		4. pre-S1+pre-S2 del (0.012)	4. pre-S1+pre-S2 del (nt 2855-2972, 1-54) (0.008)
11	wild-type	1. **wild-type (59.263)**	1. wild-type (59.263)
		2. **pre-S2 del (37.934)**	2. pre-S2 del (nt 1-15) (36.325)
		3. pre-S1 del (1.940)	3. pre-S1 del (nt 3026-3205) (1.007)
		4. pre-S1+pre-S2 del (0.862)	4. pre-S1+pre-S2 del (nt 3106-3126, 1-15) (0.439)
12	1. wild-type	1. **wild-type (82.957)**	1. wild-type (82.957)
	2. pre-S1 del (nt 2854-2970)	2. **pre-S1 del (10.988)**	2. pre-S1 del (nt 2854-2970) (5.998)
		3. pre-S2 del (4.178)	3. pre-S2 del (nt 1-54) (3.649)
		4. pre-S1+pre-S2 del (1.877)	4. pre-S1+pre-S2 del (nt 2855-2872, 1-54) (0.748)

Supplementary Table 1. List of the pre-S genotyping results by TA cloning- and NGS-based analyses in 49 HBV-related HCC patients (continued)

Patient No.	TA Cloning Result[a]	NGS Result (Pre-S Deletion Type (%))[b]	NGS Result (Pre-S Deletion Region (%))[c]
13	wild-type	1. **wild-type (93.746)**	1. wild-type (93.746)
		2. **pre-S1 del (5.846)**	2. pre-S1 del (nt 3026-3205) (2.417)
		3. pre-S2 del (0.389)	3. pre-S2 del (nt 3211-3213) (0.135)
		4. pre-S1+pre-S2 del (0.018)	4. pre-S1+pre-S2 del (nt 2856-2873, 1-21) (0.004)
14	wild-type	1. **pre-S1+pre-S2 del (46.237)**	1. pre-S1+pre-S2 del (nt 2956-3126, 1-9) (24.002)
		2. **pre-S2 del (26.927)**	2. pre-S2 del (nt 1-54) (23.226)
		3. **pre-S1 del (14.368)**	3. pre-S1 del (nt 2944-3075) (5.140)
		4. **wild-type (12.467)**	4. wild-type (12.467)
15	wild-type	1. **wild-type (97.851)**	1. wild-type (97.851)
		2. pre-S1 del (2.037)	2. pre-S1 del (nt 3026-3205) (1.109)
		3. pre-S2 del (0.108)	3. pre-S2 del (nt 3211-3213) (0.049)
		4. pre-S1+pre-S2 del (0.005)	4. pre-S1+pre-S2 del (nt 2855-2872, 1-54) (0.003)
16	wild-type	1. **pre-S2 del (56.155)**	1. pre-S2 del (nt 49-54) (56.043)
		2. **wild-type (42.610)**	2. wild-type (42.610)
		3. pre-S1+pre-S2 del (0.718)	3. pre-S1+pre-S2 del (nt 3105-3126, 49-53) (0.251)
		4. pre-S1 del (0.516)	4. pre-S1 del (nt 3105-3128) (0.103)
17	wild-type	1. **pre-S1 del (76.151)**	1. pre-S1 del (nt 3110-3127) (16.407)
		2. **wild-type (19.034)**	2. wild-type (19.034)
		3. pre-S2 del (4.624)	3. pre-S2 del (nt 1-54) (4.081)
		4. pre-S1+pre-S2 del (0.192)	4. pre-S1+pre-S2 del (nt 2855-2872, 2897-2923, 1-54) (0.051)
18	wild-type	1. **wild-type (98.490)**	1. wild-type (98.490)
		2. pre-S1 del (1.290)	2. pre-S1 del (nt 3110-3166) (0.209)
		3. pre-S2 del (0.215)	3. pre-S2 del (nt 1-12) (0.087)
		4. pre-S1+pre-S2 del (0.005)	4. pre-S1+pre-S2 del (nt 2855-2872, 1-54) (0.001)

Supplementary Table 1. List of the pre-S genotyping results by TA cloning- and NGS-based analyses in 49 HBV-related HCC patients (continued)

Patient No.	TA Cloning Result[a]	NGS Result (Pre-S Deletion Type (%))[b]	NGS Result (Pre-S Deletion Region (%))[c]
19	wild-type	1. **wild-type (98.964)**	1. wild-type (98.964)
		2. pre-S1 del (0.943)	2. pre-S1 del (nt 2854-2970) (0.125)
		3. pre-S2 del (0.084)	3. pre-S2 del (nt 1-9) (0.028)
		4. pre-S1+pre-S2 del (0.009)	4. pre-S1+pre-S2 del (nt 2954-3097, 1-13) (0.009)
20	wild-type	1. **wild-type (97.781)**	1. wild-type (97.781)
		2. pre-S1 del (2.099)	2. pre-S1 del (nt 3026-3205) (1.099)
		3. pre-S2 del (0.112)	3. pre-S2 del (nt 3211-3216) (0.055)
		4. pre-S1+pre-S2 del (0.008)	4. pre-S1+pre-S2 del (nt 3026-3205, 43-138) (0.002)
21	wild-type	1. **wild-type (94.701)**	1. wild-type (94.701)
		2. **pre-S1 del (5.086)**	2. pre-S1 del (nt 3026-3205) (2.765)
		3. pre-S2 del (0.137)	3. pre-S2 del (nt 51-143) (0.030)
		4. pre-S1+pre-S2 del (0.077)	4. pre-S1+pre-S2 del (nt 2854-2997, 25-128) (0.014)
22	wild-type	1. **wild-type (92.291)**	1. wild-type (92.291)
		2. pre-S1 del (4.092)	2. pre-S1 del (nt 2854-2970) (0.650)
		3. pre-S2 del (3.038)	3. pre-S2 del (nt 1-54) (2.641)
		4. pre-S1+pre-S2 del (0.578)	4. pre-S1+pre-S2 del (nt 2855-2872, 1-54) (0.435)
23	wild-type	1. **wild-type (69.001)**	1. wild-type (69.001)
		2. **pre-S1 del (20.530)**	2. pre-S1 del (nt 3110-3127) (4.779)
		3. **pre-S2 del (9.463)**	3. pre-S2 del (nt 1-54) (8.226)
		4. pre-S1+pre-S2 del (1.006)	4. pre-S1+pre-S2 del (nt 2855-2872, 1-18, 26-59) (0.259)
24	wild-type	1. **wild-type (50.938)**	1. wild-type (50.938)
		2. **pre-S1 del (19.760)**	2. pre-S1 del (nt 2854-2970) (5.470)
		3. **pre-S1+pre-S2 del (15.021)**	3. pre-S1+pre-S2 del (nt 2855-2872, 1-54) (12.421)
		4. **pre-S2 del (14.280)**	4. pre-S2 del (nt 1-54) (13.606)

Supplementary Table 1. List of the pre-S genotyping results by TA cloning- and NGS-based analyses in 49 HBV-related HCC patients (continued)

Patient No.	TA Cloning Result[a]	NGS Result (Pre-S Deletion Type (%))[b]	NGS Result (Pre-S Deletion Region (%))[c]
25	wild-type	1. **wild-type (96.830)**<br>2. pre-S1 del (1.728)<br>3. pre-S2 del (1.341)<br>4. pre-S1+pre-S2 del (0.102)	1. wild-type (96.830)<br>2. pre-S1 del (nt 3110-3127) (0.382)<br>3. pre-S2 del (nt 7-18, 23-40, 43-54) (0.577)<br>4. pre-S1+pre-S2 del (nt 2855-2872, 1-16) (0.041)
26	wild-type	1. **wild-type (98.571)**<br>2. pre-S1 del (1.173)<br>3. pre-S2 del (0.178)<br>4. pre-S1+pre-S2 del (0.078)	1. wild-type (98.571)<br>2. pre-S1 del (nt 3106-3129) (0.242)<br>3. pre-S2 del (nt 2-149) (0.043)<br>4. pre-S1+pre-S2 del (nt 2854-2985, 27-149) (0.014)
27	wild-type	1. **wild-type (96.915)**<br>2. pre-S1 del (2.701)<br>3. pre-S2 del (0.364)<br>4. pre-S1+pre-S2 del (0.020)	1. wild-type (96.915)<br>2. pre-S1 del (nt 3026-3205) (1.755)<br>3. pre-S2 del (nt 25-54) (0.148)<br>4. pre-S1+pre-S2 del (nt 2854-2988, 45-149) (0.004)
28	wild-type	1. **wild-type (98.069)**<br>2. pre-S1 del (1.263)<br>3. pre-S2 del (0.609)<br>4. pre-S1+pre-S2 del (0.059)	1. wild-type (98.069)<br>2. pre-S1 del (nt 3103-3126) (0.253)<br>3. pre-S2 del (nt 1-15) (0.535)<br>4. pre-S1+pre-S2 del (nt 2854-2996, 44-144) (0.015)
29	wild-type	1. **wild-type (69.571)**<br>2. **pre-S2 del (18.590)**<br>**3. pre-S1+pre-S2 del (8.224)**<br>4. pre-S1 del (3.615)	1. wild-type (69.571)<br>2. pre-S2 del (nt 1-54) (18.482)<br>3. pre-S1+pre-S2 del (nt 2855-2872, 1-54) (7.712)<br>4. pre-S1 del (nt 2855-2872) (2.674)
30	1. wild-type<br>2. pre-S2 del (nt 1-54)	1. **wild-type (39.553)**<br>2. **pre-S1 del (22.574)**<br>3. **pre-S2 del (22.250)**<br>4. **pre-S1+pre-S2 del (15.622)**	1. wild-type (39.553)<br>2. pre-S1 del (nt 2855-2872) (9.874)<br>3. pre-S2 del (nt 1-54) (16.708)<br>4. pre-S1+pre-S2 del (nt 2855-2872, 1-54) (13.250)

Supplementary Table 1. List of the pre-S genotyping results by TA cloning- and NGS-based analyses in 49 HBV-related HCC patients (continued)

Patient No.	TA Cloning Result[a]	NGS Result (Pre-S Deletion Type (%))[b]	NGS Result (Pre-S Deletion Region (%))[c]
31	wild-type	1. **pre-S2 del (41.477)**	1. pre-S2 del (nt 1-54) (24.620)
		2. **pre-S1+pre-S2 del (39.126)**	2. pre-S1+pre-S2 del (nt 2855-2872, 1-54) (26.451)
		3. **wild-type (12.348)**	3. wild-type (12.348)
		4. **pre-S1 del (7.048)**	4. pre-S1 del (nt 2855-2872) (4.978)
32	wild-type	1. **pre-S1+pre-S1 del (45.703)**	1. pre-S1+pre-S1 del (nt 2855-2872, 1-18, 26-59) (20.608)
		2. **pre-S2 del (29.458)**	2. pre-S2 del (nt 1-18, 26-59) (15.861)
		3. **wild-type (14.926)**	3. wild-type (14.926)
		4. **pre-S1 del (9.913)**	4. pre-S1 del (nt 2855-2872) (6.398)
33	1. wild-type	1. **wild-type (65.687)**	1. wild-type (65.687)
	2. pre-S2 del (nt 1-54)	2. **pre-S2 del (15.691)**	2. pre-S2 del (nt 1-54) (11.915)
		**3. pre-S1+pre-S2 del (11.160)**	3. pre-S1+pre-S2 del (nt 2855-2872, 1-54) (8.302)
		4. **pre-S1 del (7.461)**	4. pre-S1 del (nt 2854-2970) (2.589)
34	1. wild-type	1. **wild-type (30.944)**	1. wild-type (30.944)
	2. pre-S1 del (nt 2855-2872)	2. **pre-S2 del (30.409)**	2. pre-S2 del (nt 1-54) (19.525)
		3. **pre-S1+pre-S2 del (29.105)**	3. pre-S1+pre-S2 del (nt 2855-2872, 1-54) (23.945)
		4. **pre-S1 del (9.542)**	4. pre-S1 del (nt 2855-2872) (8.283)
35	1. wild-type	1. **pre-S2 del (42.909)**	1. pre-S2 del (nt 1-54) (38.091)
	2. pre-S1 del (nt 2855-2872)	2. **pre-S1+pre-S2 del (27.915)**	2. pre-S1+pre-S2 del (2855-2872, 1-54) (25.090)
		3. **wild-type (17.564)**	3. wild-type (17.564)
		4. **pre-S1 del (11.612)**	4. pre-S1 del (nt 2855-2872) (8.072)
36	wild-type	1. **pre-S1 del (56.479)**	1. pre-S1 del (nt 2854-3147) (51.379)
		2. **wild-type (29.622)**	2. wild-type (29.622)
		3. **pre-S2 del (10.198)**	3. pre-S2 del (nt 1-54) (6.759)
		4. pre-S1+pre-S2 del (3.701)	4. pre-S1+pre-S2 del (nt 2855-2872, 1-54) (2.809)

Supplementary Table 1. List of the pre-S genotyping results by TA cloning- and NGS-based analyses in 49 HBV-related HCC patients (continued)

Patient No.	TA Cloning Result[a]	NGS Result (Pre-S Deletion Type (%))[b]	NGS Result (Pre-S Deletion Region (%))[c]
37	wild-type	1. **pre-S2 del (94.816)**	1. pre-S2 del (nt 1-54) (94.697)
		2. **wild-type (5.049)**	2. wild-type (5.049)
		3. pre-S1+pre-S2 del (0.070)	3. pre-S1+pre-S2 del (nt 3152-3202, 1-54) (0.023)
		4. pre-S1 del (0.066)	4. pre-S1 del (nt 3108-3137) (0.011)
38	wild-type	1. **wild-type (65.077)**	1. wild-type (65.077)
		2. **pre-S2 del (28.468)**	2. pre-S2 del (nt 1-54) (17.044)
		3. pre-S1 del (4.516)	3. pre-S1 del (nt 2855-2872) (1.096)
		4. pre-S1+pre-S2 del (1.940)	4. pre-S1+pre-S2 del (nt 2855-2872, 1-54) (1.471)
39	wild-type	1. **wild-type (50.816)**	1. wild-type (50.816)
		2. **pre-S1 del (20.937)**	2. pre-S1 del (nt 2856-2969) (13.372)
		3. **pre-S2 del (18.426)**	3. pre-S2 del (nt 1-54) (8.012)
		4. **pre-S1+pre-S2 del (9.822)**	4. pre-S1+pre-S2 del (nt 2855-2872, 1-54) (2.746)
40	pre-S1+pre-S2 del (nt 2954-3097, 1-15)	1. **pre-S1+pre-S2 del (59.573)**	1. pre-S1+pre-S2 del (nt 2954-3097, 1-15) (31.264)
		2. **pre-S2 del (20.595)**	2. pre-S2 del (nt 1-15) (12.097)
		3. **pre-S1 del (12.197)**	3. pre-S1 del (nt 2954-3097) (5.839)
		4. **wild-type (7.634)**	4. wild-type (7.634)
41	pre-S2 del (nt 1-15)	1. **wild-type (42.161)**	1. wild-type (42.161)
		2. **pre-S1 del (36.122)**	2. pre-S1 del (nt 2941-3204) (22.006)
		3. **pre-S2 del (17.277)**	3. pre-S2 del (nt 1-15) (11.316)
		4. pre-S1+pre-S2 del (4.440)	4. pre-S1+pre-S2 del (nt 2855-2971, 1-15) (1.761)
42	pre-S1 del (nt 2855-2872)	1. **wild-type (57.089)**	1. wild-type (57.089)
		2. **pre-S1 del (32.074)**	2. pre-S1 del (nt 2855-2872) (11.018)
		**3. pre-S2 del (8.056)**	3. pre-S2 del (nt 1-15) (6.190)
		4. pre-S1+pre-S2 del (2.781)	4. pre-S1+pre-S2 del (nt 2855-2872, 1-18, 26-59) (0.956)

Supplementary Table 1. List of the pre-S genotyping results by TA cloning- and NGS-based analyses in 49 HBV-related HCC patients (continued)

Patient No.	TA Cloning Result[a]	NGS Result (Pre-S Deletion Type (%))[b]	NGS Result (Pre-S Deletion Region (%))[c]
43	1. wild-type	1. **wild-type (48.249)**	1. wild-type (48.249)
	2. pre-S1 del (nt 2856-2975)	2. **pre-S1 del (39.020)**	2. pre-S1 del (nt 2856-2975) (31.989)
		3. **pre-S1+pre-S2 del (6.890)**	3. pre-S1+pre-S2 del (nt 2855-2972, 1-54) (3.062)
		4. **pre-S2 del (5.841)**	4. pre-S2 del (nt 1-54) (5.259)
44	1. wild-type	1. **wild-type (49.088)**	1. wild-type (49.088)
	2. pre-S1 del (nt 2854-2970)	2. **pre-S2 del (28.126)**	2. pre-S2 del (nt 1-54) (17.917)
	3. pre-S1+pre-S2 del	3. **pre-S1 del (12.973)**	3. pre-S1 del (nt 2854-2970) (9.118)
	(nt 2854-2970, 1-54)	4. **pre-S1+pre-S2 del (9.812)**	4. pre-S1+pre-S2 del (nt 2854-2970, 1-54) (5.350)
45	1. wild-type	1. **wild-type (60.711)**	1. wild-type (60.711)
	2. pre-S1 del (nt 2854-2970)	2. **pre-S1 del (32.370)**	2. pre-S1 del (nt 2854-2970) (26.909)
		3. pre-S2 del (4.244)	3. pre-S2 del (nt 24-50) (2.041)
		4. pre-S1+pre-S2 del (2.674)	4. pre-S1+pre-S2 del (nt 2854-2970, 1-54) (0.989)
46	wild-type	1. **wild-type (66.570)**	1. wild-type (66.570)
		2. **pre-S1 del (25.384)**	2. pre-S1 del (nt 2854-2970) (15.571)
		3. **pre-S2 del (5.586)**	3. pre-S2 del (nt 24-50) (2.674)
		4. pre-S1+pre-S2 del (2.459)	4. pre-S1+pre-S2 del (nt 2854-2970, 24-50) (0.960)
47	wild-type	1. **wild-type (59.772)**	1. wild-type (59.772)
		2. **pre-S1 del (27.161)**	2. pre-S1 del (nt 2854-2970) (17.860)
		3. **pre-S2 del (7.973)**	3. pre-S2 del (nt 1-54) (3.934)
		4. **pre-S1+pre-S2 del (5.094)**	4. pre-S1+pre-S2 del (nt 2854-2973, 1-54) (2.304)
48	1. wild-type	1. **wild-type (54.293)**	1. wild-type (54.293)
	2. pre-S1+pre-S2 del	2. **pre-S2 del (18.740)**	2. pre-S2 del (nt 1-54) (14.741)
	(nt 2855-2872, 1-54)	3. **pre-S1 del (17.510)**	3. pre-S1 del (nt 2855-2872) (6.495)
		4. **pre-S1+pre-S2 del (9.457)**	4. pre-S1+pre-S2 del (nt 2855-2872, 1-54) (7.009)

Supplementary Table 1. List of the pre-S genotyping results by TA cloning- and NGS-based analyses in 49 HBV-related HCC patients (continued)

Patient No.	TA Cloning Result[a]	NGS Result (Pre-S Deletion Type (%))[b]	NGS Result (Pre-S Deletion Region (%))[c]
49	1. wild-type<br>2. pre-S1+pre-S2 del<br>(nt 2855-2872, 1-54)	1. **wild-type (52.661)**<br>2. **pre-S2 del (25.093)**<br>3. **pre-S1+pre-S2 del (11.424)**<br>4. **pre-S1 del (10.821)**	1. wild-type (52.661)<br>2. pre-S2 del (nt 1-54) (18.682)<br>3. pre-S1+pre-S2 del (nt 2855-2872, 1-54) (8.344)<br>4. pre-S1 del (nt 2855-2872) (2.817)

[a]All the PCR bands visualized in agarose gel were analyzed and listed in descending size order.

[b]The total frequency of pre-S gene DNA in each type of pre-S deletion was shown in descending order.

[c]The pre-S gene DNA with the highest frequency in each type of pre-S deletion was shown.

[d]The pre-S deletion type above the cut-off percentage was shown in bold.

Abbreviations: nt, nucleotide; del, deletion; n, number.
